# Macular retinal and choroidal thickness profile in patients with thyroid‐associated orbitopathy compared to healthy individuals: A cross‐sectional study

**DOI:** 10.1002/hsr2.1604

**Published:** 2023-10-03

**Authors:** Aliakbar Sabermoghaddam, Mojtaba Abrishami, Mehrdad Motamed Shariati, Zeinab Salahi

**Affiliations:** ^1^ Eye Research Center Mashhad University of Medical Sciences Mashhad Iran

**Keywords:** macula, optical coherence tomogram, thyroid associated orbitopathy

## Abstract

**Background and Aims:**

To evaluate the macular thickness profile and central subfoveal choroidal thickness in patients with thyroid‐associated orbitopathy (TAO) compared to healthy subjects.

**Methods:**

We used the convenience sampling method and divided all participants into the patients and control groups. Based on the clinical activity score (CAS) in the first examination, the patient group was divided to two subgroups: the patients with CAS < 3 and the patients with CAS ≥ 3. Complete ophthalmologic examinations and optical coherence tomography imaging were performed for all participants.

**Results:**

The mean ± SD of central choroidal thickness was 277 ± 76.58 microns for the control and 326.07 ± 56.574 micron for the patient group which was statistically significant (*p* = 0.003). We also found that the parafoveal inner thickness is significantly lower in patients compared to healthy subjects (*p* = 0.02). A comparison of neuro‐structural data between the two subgroups of patients showed a significant difference in central choroidal thickness (*p* = 0.05).

**Conclusion:**

This study showed that central choroidal thickness in patients with CAS ≥ 3 compared to those with CAS < 3 and also in the patient group compared to healthy individuals have a significantly increasing trend.

## 1 INTRODUCTION

Thyroid associated orbitopathy (TAO) is one of the frequent complications in patients with hyperthyroidism which occurs in 25%−50% of graves cases, but it also occurs in hypothyroid and euthyroid patients.^1^ Although autoimmune mechanisms have been postulated, the exact pathophysiology is unknown. TAO starts with an active inflammatory phase with an estimated duration of 6−36 months. Over time, this inflammation subsides and a chronic fibrotic phase happens which may accompany visual function decrement.^2^ Theoretically, the best time for treatment is the inflammatory phase. Starting treatment in this phase can reduce complications and the need for surgeries in the future.^3^ Several risk factors have been proposed for TAO including positive family history, smoking, older age, female gender, and hyperthyroidism treatment. It seems that the environmental factors in a genetically susceptible individual lead to the occurrence of the disease.^4^ Common manifestations of TAO include lid retraction, chemosis, proptosis, dry eye, exposure‐keratopathy, strabismus due to restrictive myopathy, and optic neuropathy. These signs and symptoms negatively affect the patients' quality of life and even lead to an irreversible decrease in vision.^5^

Dysthyroid optic neuropathy (DON) occurs in 3%−7% of patients with TAO.^6^ In the pathogenesis of DON, three main factors have been proposed: mechanical, inflammatory, and vascular factors. Orbital fibroblasts' activation by different stimulators such as the insulin‐like growth factor‐1 (IGF‐1) and anti‐thyroid stimulating hormone antibodies, leads to cell proliferation, adipogenesis, and production of excessive hyaluronic acid. These changes besides the extraocular muscle (EOM) enlargement can cause a compressive effect on the optic nerve. This compartment syndrome causes disturbances in axonal flow and ischemic injuries, which finally leads to axonal loss.^6^, ^7^ Most of the optic nerve axons are related to the macular ganglion cells. Clinical evaluation of optic nerve function is necessary for all patients with TAO. Assessment of visual acuity, color vision, contrast sensitivity, visual field, and visual evoked potential are helpful.^7^, ^8^ Furthermore, an orbital CT scan is useful to detect optic nerve crowding at the orbital apex, EOM enlargement, and superior ophthalmic vein enlargement. The disturbances in venous drainage can cause congestion of the choroidal vessels, resulting in an increase in choroidal thickness as well as possible ischemic changes in the outer retina. The timing of the diagnosis of axonal injury is very important because early detection can prevent irreversible damage.^8^, ^9^

Optical coherence tomography (OCT) is an imaging modality for the evaluation of macular thickness profiles. In addition to being safe, easy, and accessible, with this modality, the subfoveal choroidal thickness could be assessed.^10^

This study is proposed to evaluate the macular thickness profile changes in patients with TAO compared to healthy people.

## 2 METHODS

### 2.1 Participants

In this cross‐sectional study, the macular thickness profile in TAO patients were compared to healthy individuals. Inclusion criteria for patients group consist of at least two items of concurrent or recently treated immune‐related thyroid dysfunction, typical ocular signs, and radiographic evidence of thyroid eye disease. Equal number were selected from healthy volunteers.

Both groups were matched for age and gender. Exclusion criteria were: previous ocular surgeries in the recent 6 months, intraocular pressure equal or more than 20 mmHg or any history of glaucoma, individuals younger than 18, refractive error more than 3 diopters of spherical equivalent, any diseases that can affect the choroidal thickness and neurovasculature of the retina such as uveitis, diabetes mellitus, pregnancy, breast feeding, and consumption of oral contraceptives.

### 2.2 Examinations

The Muritz clinical activity score (CAS) system^11^ Used to assess the activity of TAO. At the first examination, it includes seven items including spontaneous orbital pain, gaze‐evoked orbital pain, eyelid swelling, eyelid erythema, conjunctival redness, chemosis, and inflammation of the caruncle or plica. Each item is rated one (if that item is positive) or zero. A total score of three or more is considered an active disease. Based on the CAS in the first examination, The patients divided in two subgroups: the patients with CAS < 3 and the patients with CAS ≥ 3. All participants underwent the following ocular examinations at the “…”: best‐corrected visual acuity (BCVA) measurement with thumbing E chart, slit‐lamp biomicroscopy, Goldmann applanation tonometry, and complete fundus examination (using a+90D condensing lens) after pupil dilatation with a tropicamide eye drop.

### 2.3 Imagings

The following imaging was performed for all participants at baseline:

1. Macular OCT (AngioVueRTVue XR Avanti; Optovue software version: 2018.0.0.18) with 3 × 3 and 6 × 6 mm scan size. The foveal thickness profile was defined as the thickness profile in a 1 mm diameter circle centered on the center of the fovea. The parafoveal region was defined as the ring occupying the area between the foveal area and the 2.5 × 2.5 mm area centered on the foveal center. The perifoveal region was defined as the outer ring between the outer border of the parafovea and the 3.5 × 3.5 mm diameter on the foveal center. Total retinal thickness was defined as the distance between the internal limiting membrane (ILM) and RPE.
2. Enhanced depth imaging OCT (EDI‐OCT) of the macula to measure the subfoveal choroidal thickness. Subfoveal choroidal thickness was defined as the thickness from the Bruch's membrane to the sclerochoroidal junction.
3. The OptoVue glaucoma protocol was employed to measure the macular ganglion cell complex (GCC) thickness, which is defined as the distance between ILM and the outer border of the inner plexiform layer.

Images were taken without any pharmacologic mydriasis and after 3−5 min of rest. All measurements were taken at 8−12 a.m. Any images with a quality index below 6/10 were discarded.

Macular thickness profile includes foveal thickness profile (foveal full‐thickness, foveal inner thickness, foveal outer thickness, and central subfoveal choroidal thickness), parafoveal thickness profile (parafoveal full‐thickness, parafoveal inner thickness, parafoveal outer thickness), perifoveal thickness profile (perifoveal full‐thickness, perifoveal inner thickness, perifoveal outer thickness), and average GCC were analyzed and compared between the groups.

### 2.4 Statistical analysis

Statistical Package for Social Sciences (SPSS) software version 22 (IBM SPSS Statistics; IBM Corporation) was used for data analysis. Moreover, Shapiro−Wilk test to analysis of data distribution, *χ* ^2^ test to investigate the corelation between qualitative variables, independent *t*‐test or it's nonparametric equivalents to compare the quantitative variables, and central indices or indices of dispersion for analysis of demographic data were employed.

The characteristics of the subjects are described by descriptive statistical methods including central indices and indices of dispersion. We used the *χ* ^2^ test to investigate the relationship between the qualitative variables and the independent samples *t*‐test or its nonparametric equivalent to compare the quantitative variables between the groups. In all calculations, *p* < 0.05 was considered a significant level.

## 3 RESULTS

Eighty–five cases, including 30 healthy individuals and 55 TAO participated in our study. All thepatientswere euthyroid according to the opinion of the endocrinologist. At the first examination, 32 of 55 patient's had CAS < 3 and 23 patients had CAS ≥ 3. In this study, we analyzed the data from the right eye of each participant. Based on the Shapiro−Wilk test, the distribution of macular thickness parameters was normal. Descriptive statistics of age, gender, and smoking were summarized in Table 1. There was no statistically significant difference between the three groups in age, gender, and smoking. All of the subjects in both groups had a BCVA of 10/10, and there were no significant ocular conditions with potential impacts on retinal and choroidal neurovasculature. The mean ± SD for intraocular pressure was 11.77 ± 1.41 and 11.18 ± 1.59 mmHg for the control and patient groups, respectively (*p* = 0.5).

**Table 1:** Demographics of all participants.

Variable		Control group	Patients group		*p* Value
			CAS <3	CAS ≥3	
Age (mean ± SD)		36.56 ± 2.33	44.34 ± 2.45	50.17 ± 3.21	0.23
Gender	Male (*N*)	12	7	6	0.71
	Female (*N*)	18	25	17	
Smokers (*N*)		2	7	3	0.40

Abbreviation: CAS, clinical activity score.

### 3.1 Foveal thickness profile

While central choroidal thickness was significantly higher in patients with TAO compared to healthy subjects (*p* = 0.003), foveal thickness profile evaluation indicates no difference in retinal thickness parameters in the foveal area between patients and control group (Table 2a).

**Table 2a:** Foveal thickness profile in patients and controls.

		Mean ± SD, patients	Mean ± SD, controls	*p* Value
Foveal thickness profile (micron)	Full thickness fovea	260.15 ± 71.05	250.30 ± 17.66	0.459
	Central choroidal thickness	326.07 ± 56.74	277 ± 76.58	0.003*
	Inner thickness fovea	69.84 ± 20.33	69.00 ± 10.26	0.834
	Outer thickness fovea	190.27 ± 52.90	181.43 ± 11.81	0.370

* Considered statistically significant.

A comparison of neuro‐structural data between the two subgroups of patients showed a significant difference in central choroidal thickness (*p* = 0.05). There was no statistically significant difference in other foveal thickness parameters between the two subgroups of patients (Table 2b).

**Table 2b:** Comparison of foveal thickness profile between the two subgroups of patients.

		Mean ± SD, CAS ≥3	Mean ± SD, CAS <3	*p* Value
Foveal thickness profile (micron)	Full thickness fovea	261.3 ± 47.16	259.31 ± 94.93	0.91
	Central choroidal thickness	292.78 ± 93.81	256.35 ± 34.45	0.05*
	Inner thickness fovea	71.30 ± 16.17	68.78 ± 23.06	0.65
	Outer thickness fovea	190.09 ± 35.44	190.41 ± 63.12	0.98

Abbreviation: CAS, clinical activity score.

* Considered statistically significant.

### 3.2 Parafoveal thickness profile

The mean ± SD of parafoveal inner thickness was 131.57 ± 7.52 micron for the control and 126.15 ± 13.9 micron for the patient group which was statistically significant (*p* = 0.02). Furthermore, a significant decrement was observed in parafoveal inner retinal thickness at superior and temporal quadrants between patients and the control group. We found no significant difference between these two groups for the other thickness parameters of the parafovea (Table 3a).

**Table 3a:** Parafoveal thickness profile in patients and controls.

		Mean ± SD, patients	Mean ± SD, controls	*p* Value
Parafoveal thickness profile (micron)	Full thickness parafovea	317.60 ± 42.68	318.20 ± 16.63	0.941
	Full thickness parafovea temporal	305.58 ± 34.49	309.27 ± 17.46	0.586
	Full thickness parafovea superior	324.84 ± 56.54	328.83 ± 16.89	1.000
	Full thickness parafovea nasal	320.24 ± 53.41	319.83 ± 17.26	0.968
	Full thickness parafovea inferior	316.91 ± 33.82	319.17 ± 17.97	0.735
	Inner thickness parafovea	126.15 ± 13.9	131.57 ± 7.52	0.022*
	Inner thickness parafovea temporal	116.73 ± 11.88	124.83 ± 8.87	0.002*
	Inner thickness parafovea superior	130.47 ± 15.31	137.13 ± 8.64	0.012*
	Inner thickness parafovea nasal	126.05 ± 17.01	131.40 ± 8.70	0.059
	Inner thickness parafovea inferior	128.38 ± 15.58	132.63 ± 8.82	0.112
	Outer thickness parafovea	191.91 ± 34.45	186.73 ± 12.84	0.431
	Outer thickness parafovea temporal	188.58 ± 27.66	184.40 ± 12.31	0.435
	Outer thickness parafovea superior	194.71 ± 50.36	187.67 ± 14.35	0.457
	Outer thickness parafovea nasal	194.09 ± 42.61	188.43 ± 13.24	0.482
	Outer thickness parafovea inferior	188.93 ± 23.41	186.67 ± 14.83	0.634

* Considered statistically significant.

Although the differences in parafoveal inner thickness between the two subgroups of patients were significant in the temporal and superior quadrants, the difference in the mean parafoveal inner thickness was not significant between the two subgroups. There were no statistically significant differences in all other neuro‐structural parameters of the parafovea between the two subgroups of patients (Table 3b).

**Table 3b:** Comparison of parafoveal thickness profile between the two subgroups of patients.

		Mean ± SD, CAS <3	Mean ± SD, CAS ≥3	*p* Value
Parafoveal thickness profile (micron)	Full thickness parafovea	320.59 ± 49.52	313.43 ± 33.52	0.545
	Full thickness parafovea temporal	307.13 ± 31.34	303.43 ± 39.06	0.699
	Full thickness parafovea superior	330.09 ± 60.41	317.52 ± 51.07	0.421
	Full thickness parafovea nasal	326.16 ± 65.66	312 ± 28.40	0.337
	Full thickness parafovea inferior	319.19 ± 40.10	313.74 ± 22.88	0.561
	Inner thickness parafovea	128.81 ± 14.00	122.43 ± 13.17	0.094
	Inner thickness parafovea temporal	120.22 ± 10.37	111.87 ± 12.34	0.009*
	Inner thickness parafovea superior	134.06 ± 15.53	125.48 ± 13.81	0.039*
	Inner thickness parafovea nasal	129.81 ± 16.05	120.83 ± 17.26	0.052
	Inner thickness parafovea inferior	130.88 ± 16.65	124.91 ± 13.55	0.164
	Outer thickness parafovea	192.03 ± 37.80	191.74 ± 30.01	0.976
	Outer thickness parafovea temporal	186.81 ± 23.30	191.04 ± 33.20	0.581
	Outer thickness parafovea superior	196.22 ± 51.16	192.61 ± 50.30	0.796
	Outer thickness parafovea nasal	196.22 ± 53.04	191.13 ± 21.86	0.666
	Outer thickness parafovea inferior	188.41 ± 26.86	189.65 ± 18.09	0.848

Abbreviation: CAS, clinical activity score.

* Considered statistically significant.

### 3.3 Perifoveal thickness profile

There was no significant difference in perifoveal thickness profile between the two groups of patient and control (Table 4a) and also between the two subgroups of patients based on CAS score (Table 4b).

**Table 4a:** Perifoveal thickness profile in patients and controls.

		Mean ± SD, patients	Mean ± SD, controls	*p* Value
Perifoveal thickness profile (micron)	Full thickness perifovea	288.78 ± 30.59	290.87 ± 16.21	0.730
	Full thickness perifovea temporal	276.15 ± 28.73	278.53 ± 15.66	0.674
	Full thickness perifovea superior	293.22 ± 48.11	293.53 ± 17.13	0.972
	Full thickness perifovea nasal	306.96 ± 33.88	308.13 ± 17.72	0.861
	Full thickness perifovea inferior	281.04 ± 23.16	283.07 ± 17.25	0.675
	Inner thickness perifovea	111.80 ± 11.90	114.97 ± 7.69	0.193
	Inner thickness perifovea temporal	105.60 ± 12.25	108.77 ± 7.17	0.137
	Inner thickness perifovea superior	111.51 ± 14.07	114.43 ± 7.85	0.296
	Inner thickness perifovea nasal	123.40 ± 14.05	125.07 ± 9.71	0.565
	Inner thickness perifovea inferior	109.51 ± 11.36	111.50 ± 9.36	0.415
	Outer thickness perifovea	176.36 ± 22.72	175.90 ± 11.52	0.917
	Outer thickness perifovea temporal	170.67 ± 20.31	169.83 ± 10.67	0.834
	Outer thickness perifovea superior	181.42 ± 38.44	179.03 ± 13.25	0.743
	Outer thickness perifovea nasal	183.42 ± 25.54	183.13 ± 13.65	0.955
	Outer thickness perifovea inferior	171.09 ± 14.82	171.67 ± 11.36	0.854

**Table 4b:** Comparison of perifoveal thickness profile between the two subgroups of patients.

		Mean ± SD, CAS <3	Mean ± SD, CAS ≥3	*p* Value
Perifoveal thickness profile (micron)	Full thickness perifovea	293.50 ± 31.64	282.22 ± 28.24	0.180
	Full thickness perifovea temporal	277.59 ± 21.48	274.13 ± 36.99	0.663
	Full thickness perifovea superior	301.09 ± 52.28	282.26 ± 40.18	0.154
	Full thickness perifovea nasal	311.59 ± 39.30	300.52 ± 23.82	0.235
	Full thickness perifovea inferior	283.81 ± 23.91	277.17 ± 22.02	0.299
	Inner thickness perifovea	115.59 ± 10.59	106.52 ± 11.80	0.004*
	Inner thickness perifovea temporal	108.03 ± 10.24	102.22 ± 14.14	0.083
	Inner thickness perifovea superior	116.16 ± 12.61	105.04 ± 13.66	0.003*
	Inner thickness perifovea nasal	126.25 ± 12.60	119.43 ± 15.25	0.076
	Inner thickness perifovea inferior	112 ± 10.04	106.04 ± 12.37	0.054
	Outer thickness perifovea	177.31 ± 24.44	175.04 ± 20.54	0.719
	Outer thickness perifovea temporal	169.34 ± 13.92	172.52 ± 27.09	0.572
	Outer thickness perifovea superior	184.44 ± 42.67	177.22 ± 32.07	0.497
	Outer thickness perifovea nasal	184.72 ± 31.32	181.61 ± 14.57	0.660
	Outer thickness perifovea inferior	171.31 ± 16.06	170.78 ± 13.24	0.897

Abbreviation: CAS, clinical activity score.

* Considered statistically significant.

### 3.4 GCC

Although the mean GCC in the patient group was lower than the control group, this difference was not significant (Table 5a).

**Table 5a:** GCC in patients and controls.

		Mean ± SD, patients	Mean ± SD, controls	*p* Value
GCC (micron)	Average GCC	96.04 ± 17.39	99.87 ± 6.36	0.249
		0−140	89−116	
	Superior GCC	95.53 ± 18.18	99.10 ± 6.57	0.303
		0−153	87−118	
	Inferior GCC	96.60 ± 16.58	100.60 ± 6.60	0.209
		0−127	90−114	

Abbreviation: GCC, ganglion cell complex.

A comparison of GCC between the two subgroups of patients showed no significant difference (Table 5b).

**Table 5b:** Comparison of GCC between the two subgroups of patients.

		Mean ± SD, CAS <3	Mean ± SD, CAS ≥3	*p* Value
GCC (micron)	Average GCC	95.31 ± 19.87	97.04 ± 13.55	0.719
	Superior GCC	94.28 ± 19.69	97.26 ± 16.12	0.554
	Inferior GCC	96.25 ± 20.14	97.09 ± 10.14	0.856

Abbreviations: CAS, clinical activity score; GCC, ganglion cell complex.

## 4 DISCUSSION

Concerns about ONH changes in patients with TAO have recently been addressed. Increased intra‐orbital pressure and increased EOM volume can potentially lead to optic neuropathy.^12^ Evaluation of blood flow in the orbit indicates venous stasis and increase superior ophthalmic vein diameter in these patients.^6^, ^12^ Various studies have reported that the function of the optic nerve may be affected before the clinical complaint of vision loss.^13^, ^14^ Detection of the onset of optic nerve damage can help prevent significant vision loss. Evidence suggests that although optic nerve compression is important in the pathogenesis of DON, ONH involvement due to microvascular ischemia secondary to orbital apex changes can be a potential influential factor.^9^, ^15^ In addition, major risk factors for DON include age, smoking, and diabetes; which can be associated with microvascular changes.^6^

Most of the axons of the optic nerve belong to the macular region. Due to the transparent media of the eye, which allows the evaluation of retinal neurovascular tissues, the study of retinal changes as a model in systemic diseases is a hot topic right now. This study was designed to investigate macular thickness profiles in patients with TAO compared to healthy individuals. Evaluation of thickness retinal parameters is easy, safe, readily available, and relatively inexpensive with OCT.

Our results showed a significantly higher central choroidal thickness in patients with TAO compared to healthy subjects which is compatible with previous studies. The normal choroidal thickness in healthy subjects is approximately 250−350 μm, which varies, depending on many factors, including age, sex, refractive error, and diurnal variation.^16^, ^17^ Increased superior ophthalmic venous pressure and inflammatory processes may justify the finding that the former is more acceptable. Besides, we showed that central choroidal thickness is significantly higher in patients with CAS ≥ 3 compared with those with CAS < 3 at the first examination. The relationship between the central choroidal thickness and CAS score in other studies indicates controversial results.^18^ Our findings suggest that CCT could be a potentially good candidate for monitoring the disease activity.^18^ However, the reversibility of CCT increase was not evaluated in this study. Dave et al. in a study in 2022 showed choroidal vasculature is a valuable measurement in differentiating the noninflammatory active TAO from the inactive state.^17^ These significant changes were observed while visual acuity was not reduced in patients. Based on the results of a systematic review, it seems that choroidal thickness could be a potential paraclinical biomarker for the activity of TAO, however, the heterogeneity of the studies limits the precise conclusion.^19^

We showed a significantly lower mean parafoveal inner thickness in patients with TAO than in healthy individuals. Comparison between two subgroups of patients showed a significant decrement in perifoveal inner thickness in patients with CAS ≥ 3. The thinner inner layers of the retina can be due to retrograde degeneration of ganglion axons because of compressive optic neuropathy or local circulatory disturbances of the inner retina. Despite the lower parafoveal inner thickness in patients with TAO, GCC evaluation in this study indicates no significant difference in the study groups.

This study has limitations such as small sample size and type of study design which was cross‐sectional, and short duration of follow‐up of patients with CAS ≥ 3. Besides, we did not evaluate the effects of treatment on the retinal and choroidal thickness profile in patients with TAO. Furthermore, we did not evaluate the visual fields of patients simultaneously. By performing a functional test such as perimetry, it was possible to evaluate the precedence or latency of structural and functional changes. Another limitation in this study was the lack of separate analysis of data by gender due to the small sample size. Hormonal differences between the sexes as well as vasomotor changes in women of premenopausal ages are potential confounding factors in neurovascular assessments of the retina.^20^

Evaluation of macular neurostructural changes for early detection of DON is an interesting topic. For this purpose, studies with larger sample sizes and longer follow‐up durations should be performed. It seems that only by conducting a study with cohort design and long‐term follow‐up of patients and considering the effects of treatment, it is possible to determine whether changes in choroidal thickness are temporary or not in patients with TAO.

Evaluation of central subfoveal choroidal thickness changes in patients with TAO by OCT can be helpful in monitoring the disease activity. This study showed that central choroidal thickness in patients with CAS ≥ 3 compared to those with CAS < 3 and also in the patient group compared to healthy individuals have a significant increasing trend.

## AUTHOR CONTRIBUTIONS

**Aliakbar Sabermoghaddam**: Supervision. **Mojtaba Abrishami**: Methodology. **Mehrdad Motamed Shariati**: Writing—review and editing. **Zeinab Salahi**: Writing—original draft; Writing—review and editing.

## CONFLICT OF INTEREST STATEMENT

The authors declare no conflict of interest.

## ETHICS STATEMENT

This study was in accordance with the Declaration of Helsinki, and the “…” Medical Sciences' Ethical Committee approved this study (approval number: “…” MEDICALREC.1399.260). We obtained informed consent from the patients.

## TRANSPARENCY STATEMENT

The lead author Zeinab Salahi affirms that this manuscript is an honest, accurate, and transparent account of the study being reported; that no important aspects of the study have been omitted; and that any discrepancies from the study as planned (and, if relevant, registered) have been explained.

## Data Availability

The data that support the findings of this study are available from the corresponding author upon reasonable request.
